# Metabolomics in COPD Acute Respiratory Failure Requiring Noninvasive Positive Pressure Ventilation

**DOI:** 10.1155/2017/9480346

**Published:** 2017-12-17

**Authors:** Spyridon Fortis, Elizabeth R. Lusczek, Craig R. Weinert, Greg J. Beilman

**Affiliations:** ^1^Pulmonary, Department of Medicine, Critical Care and Occupational Medicine, University of Iowa Hospital and Clinics, Iowa City, IA, USA; ^2^Critical Care and Acute Care Surgery Division, Department of Surgery, University of Minnesota, Minneapolis, MN, USA; ^3^Pulmonary and Critical Care Division, Department of Medicine, University of Minnesota, Minneapolis, MN, USA

## Abstract

We aimed to investigate whether metabolomic analysis can discriminate acute respiratory failure due to COPD exacerbation from respiratory failure due to heart failure and pneumonia. Since COPD exacerbation is often overdiagnosed, we focused on those COPD exacerbations that were severe enough to require noninvasive mechanical ventilation. We enrolled stable COPD subjects and patients with acute respiratory failure requiring noninvasive mechanical ventilation due to COPD, heart failure, and pneumonia. We excluded subjects with history of both COPD and heart failure and patients with obstructive sleep apnea and obstructive lung disease other than COPD. We performed metabolomics analysis using NMR. We constructed partial least squares discriminant analysis (PLS-DA) models to distinguish metabolic profiles. Serum (*p*=0.001, *R*^2^ = 0.397, *Q*^2^ = 0.058) and urine metabolic profiles (*p* < 0.001, *R*^2^ = 0.419, *Q*^2^ = 0.142) were significantly different between the four diagnosis groups by PLS-DA. After excluding stable COPD patients, the metabolomes of the various respiratory failure groups did not cluster separately in serum (*p*=0.2, *R*^2^ = 0.631, *Q*^2^ = 0.246) or urine (*p*=0.065, *R*^2^ = 0.602, *Q*^2^ = −0.134). However, several metabolites in the serum were reduced in patients with COPD exacerbation and pneumonia. We did not find a metabolic profile unique to COPD exacerbation, but we were able to clearly and reliably distinguish stable COPD patients from patients with respiratory failure in both serum and urine.

## 1. Introduction

COPD is the third leading cause of mortality in the USA and affects about 6% of the total population with a prevalence of more than 11.6% in people aged ≥ 65 years [1]. COPD is associated with high morbidity, high resource utilization and cost due to clinic visits, chronic therapy, and frequent hospitalizations [1, 2]. Severe acute exacerbations of COPD (AECOPD) require admission to the hospital and are responsible for up to 70% of the direct health-care costs associated with COPD [3, 4]. Five-year mortality of patients admitted to the hospital with AECOPD is between 50% and 70% which is comparable with the mortality of the four most common malignancies, lung cancer excepted [5]. Hospitalizations for AECOPD are associated with reduction in functional status and health-related quality of life [6, 7].

Early recognition and treatment of AECOPD is associated with shorter recovery time, reduces the hospitalization risk, and is associated with better health-related quality of life [8, 9]. Currently, there are no available biomarkers, and AECOPD still remains a clinical diagnosis, which may result in overdiagnosis and overtreatment [10].

Many human diseases including COPD are associated with an abnormal metabolic state. Serum and urine metabolomic profiling can discriminate patients with COPD and healthy subjects [11, 12]. A recent report showed that serum tryptophan levels decrease in patients with AECOPD [13]. Although patients with AECOPD were shown to have a unique metabolomic signature [13], it is unknown whether metabolomic analysis can discriminate AECOPD from other coexisting diseases and conditions like heart failure and pneumonia that often occur together with AECOPD and have similar symptomatology.

We aimed to compare the metabolic profile of patients with stable COPD, AECOPD, heart failure, and pneumonia and provide proof-of-concept evidence that serum and/or urine metabolomes contain biomarkers of AECOPD. Since AECOPD is based solely on clinical finding and it is often overdiagnosed, we focused on those AECOPD that were severe enough to require noninvasive positive pressure mechanical ventilation (NIPPV). Moreover, those patients have already demonstrated signs of respiratory muscle fatigue and likely altered metabolic state.

 
*Hypothesis*: Acute respiratory failure due to AECOPD that require NIPPV is characterized by a unique metabolic profile in serum and urine.

To investigate our hypothesis, we performed metabolomics analysis in the serum and urine in subjects with stable COPD from the clinic and hospitalized patients with respiratory failure that required NIPPV due to AECOPD, congestive heart failure (CHF), or pneumonia (PNA). We excluded patients that were admitted with more than one of the above diagnosis (AECOPD, CHF, and PNA), AECOPD patients with a heart failure history, CHF subjects with a COPD history, and subjects with history of both COPD and heart failure. We examined whether the metabolic profile in serum and urine can discriminate the various groups. In addition, we investigated whether the metabolome in the serum and urine can discriminate various outcomes including duration of NIPPV, length of stay, and mortality. Metabolomics analysis was performed with nuclear magnetic resonance (NMR) spectroscopy and partial least squares discriminant analysis (PLS-DA).

## 2. Methods

The study protocol was reviewed and approved (study number: 1310M44725) by the University of Minnesota Institutional Review Board, in accordance with the Code of Federal Regulations, 45 CFR 46.101(b).

### 2.1. Subject Selection

Based on the study by Wang et al. [11], the sensitivity and specificity of serum metabolomic analysis to discriminate COPD from healthy controls were 90% and 86.95%, respectively. Accordingly, we estimated a sample size of 10–15 would be sufficient to detect a difference in metabolomic analysis with a power of 80% between subjects with COPD respiratory failure and subjects with other types of respiratory failure. However, we need to emphasize that there is no accepted approach to estimate the sample size in metabolomic analysis, in part due to the unknown magnitude of the expected effect. In our study, we aimed to discriminate the metabolomic profiles in subjects with COPD respiratory failure from those of subjects with other types of respiratory failure while Wang et al. investigated the metabolomic profile of COPD and healthy subjects [11]. It was unclear a priori if similar metabolic effects would be observed.

We enrolled stable COPD subjects visiting the University of Minnesota Medical Center pulmonary clinic for a regular appointment, and patients with acute respiratory failure admitted to University of Minnesota Medical Center, Fairview Ridges Hospital, and Fairview Southdale Hospital requiring NIPPV with one of the following diagnoses: AECOPD, heart failure, or pneumonia. The characteristics of the hospitals have been described previously [14]. Briefly, the capacity of University of Minnesota Medical Center ICUs and step-down unit are 52 beds and 28 beds, respectively. Fairview Ridges Hospital ICU has a capacity of 12 beds, and Fairview Southdale Hospital ICU has a capacity of 22 beds.

Using electronic medical record, one of the investigators screened for eligible subjects in a convenient fashion Monday to Friday between 7 and 10 am.

### 2.2. Inclusion Criteria

We included subjects with the following conditions:

- *Stable COPD*: COPD patients, defined as subjects with COPD diagnosis per their pulmonologists, smoking history, and FEV1/FVC < lower limit of normal, with FEV1% predicted < 60% on stable respiratory condition per their pulmonologists that visited the pulmonary clinic.
- *Respiratory failure requiring NIPPV*: Patients with acute respiratory failure in the ICU or the “step down unit” that initiated NIPPV in the last 24 hours and had not discontinued NIPPV longer than 4 hours from the time of blood and urine sampling. Patients were categorized in the following subgroups:
  1. *AECOPD*: COPD exacerbation was defined as change in baseline dyspnea, cough, or sputum quantity or purulence, older than 40 years and a smoking history of 20 pack-years or more with known COPD, or COPD confirmed with PFTs.
  2. *CHF*: Acute decompensate (systolic or diastolic) heart failure was defined as change in baseline dyspnea with evidence of fluid overload, elevated natriuretic peptides, or known history of chronic systolic or diastolic heart failure.
  3. *PNA*: Pneumonia was defined as new infiltrate on admission chest X-ray (CXR) and symptoms or signs consistent with pneumonia: malaise, sputum production, fever (*T* > 38.0°C), and crackles in auscultation of the lung.

### 2.3. Exclusion Criteria

We excluded subjects with history of both COPD and heart failure. Patients admitted with acute respiratory failure due to more than one reason (e.g., COPD and CHF, COPD and PNA, and CHF and PNA) were excluded. Patients previously diagnosed with bronchial asthma, bronchiectasis, bronchiolitis related to systemic pathology, cystic fibrosis, obstructive sleep apnea, or upper airway obstruction were excluded.

After identifications of the eligible subjects, the investigators contacted the providers to ask permission to enroll the patients in the study. After informed written consent was obtained from the patients or legal surrogates, blood and urine were collected from the patients. Blood samples were centrifuged at 3000*g* for 15 min to extract serum. Serum was extracted, placed in a clean tube, and stored at −80°C for storage until NMR analysis. Urine samples were tested for osmolality and frozen and stored at −80°C. We retrieved age, sex, BMI, and last spirometric data from the patient's chart. We recorded patients' outcomes: duration of NIPPV, intubation, ICU length of stay, ICU mortality, hospital length of stay, hospital mortality, and discharge destination (home, long-term care facility, etc.). We collected all data prior to metabolomics analysis.

### 2.4. Metabolomics Analysis

Urine and serum samples were prepared for spectral analysis via nuclear magnetic resonance (NMR) at the Minnesota NMR Center. A portion of the metabolites present in each sample was identified and quantified as follows.

Thawed serum was filtered with a 3 kDa ultracentrifuge filter (Millipore, Billerica, MA) to remove proteins that interfere with metabolite quantification. Equal parts (250 μL each) of the filtrate were mixed with phosphate buffer, and internal standard (1 mM trimethylsilylpropionic acid in D_2_O) was added. The pH of the final solution was recorded, and the mixture was transferred to separate 5 mm NMR tubes (Wilmad-LabGlass, USA) [15].

Thawed urine (1 mL) was mixed with 0.5 mL of 0.2 M sodium phosphate buffer to control pH. The mixture was placed on ice for 10 minutes and then centrifuged at 7000 g for 10 minutes. 500 μL of the supernatant was extracted and combined with 50 μL of the internal standard 3-(trimethylsilyl)propionic acid (TSP, Sigma-Aldrich, USA) to a concentration of 1 mM. The pH of the final solution was recorded, and the mixture was transferred to separate 5 mm NMR tubes (Wilmad-LabGlass, USA).

Proton NMR spectra were obtained from both urine and serum samples with a Bruker Avance spectrometer with autosampler and 5 mm triple resonance 1H/13C/15N TXI CryoProbe with Z-gradient, running TopSpin v. 2.16 (Bruker BioSpin, Fremont, CA, USA) at 700.13 MHz. For the urine, a 1D NOESY (Nuclear Overhauser Effect Spectroscopy) pulse sequence was used to remove the water resonance. For the serum, a CPMG (Carr–Purcell–Meiboom–Gill) presaturation pulse sequence was used to control spectral line-broadening due to the presence of residual proteins.

### 2.5. Statistical Analysis

We compared subject characteristics using *t*-test, Mann–Whitney, ANOVA, or Kruskal-Wallis with post-hoc Bonferroni and Dunn's test when appropriate for continuous variables and Fischer's exact test for categorical variables.

For the metabolomics analysis, urine metabolite concentrations were divided by the osmolality (millimoles of solute per liter of urine) of the appropriate sample to correct for dilution [16]. All urine and serum metabolite concentrations were log-transformed and auto-scaled. Partial least squares discriminant analysis (PLS-DA) models were constructed to discriminate samples by cause of respiratory failure, duration of NIPPV, ICU length of stay, ICU mortality, hospital length of stay, and discharge destination. We used the R software package (http://www.r-project.org/) for all statistical analysis. PLS-DA model quality was evaluated with standard parameters (*R*^2^, *Q*^2^, and permutation *p* value), which are reported for each PLS-DA model. Generally, *R*^2^ indicates goodness-of-fit, *Q*^2^ indicates the model's predictive value, and permutation *p* value indicates whether the model is statistically significant or not (i.e., whether or not the observed separation was arrived at by chance).

## 3. Results

In the analysis, we included less than 10% of subjects screened since many patients met the exclusion criteria (they had respiratory failure from more than one underlying condition). There was no difference in any demographic variables between the various groups including age and female sex, BMI, FEV1% predicted, duration of NIPPV, intubation rates, length of ICU stay, ICU mortality, length of hospital stay, hospital mortality, or hospital discharge at home rates (Table 1).

**Figure 1:**
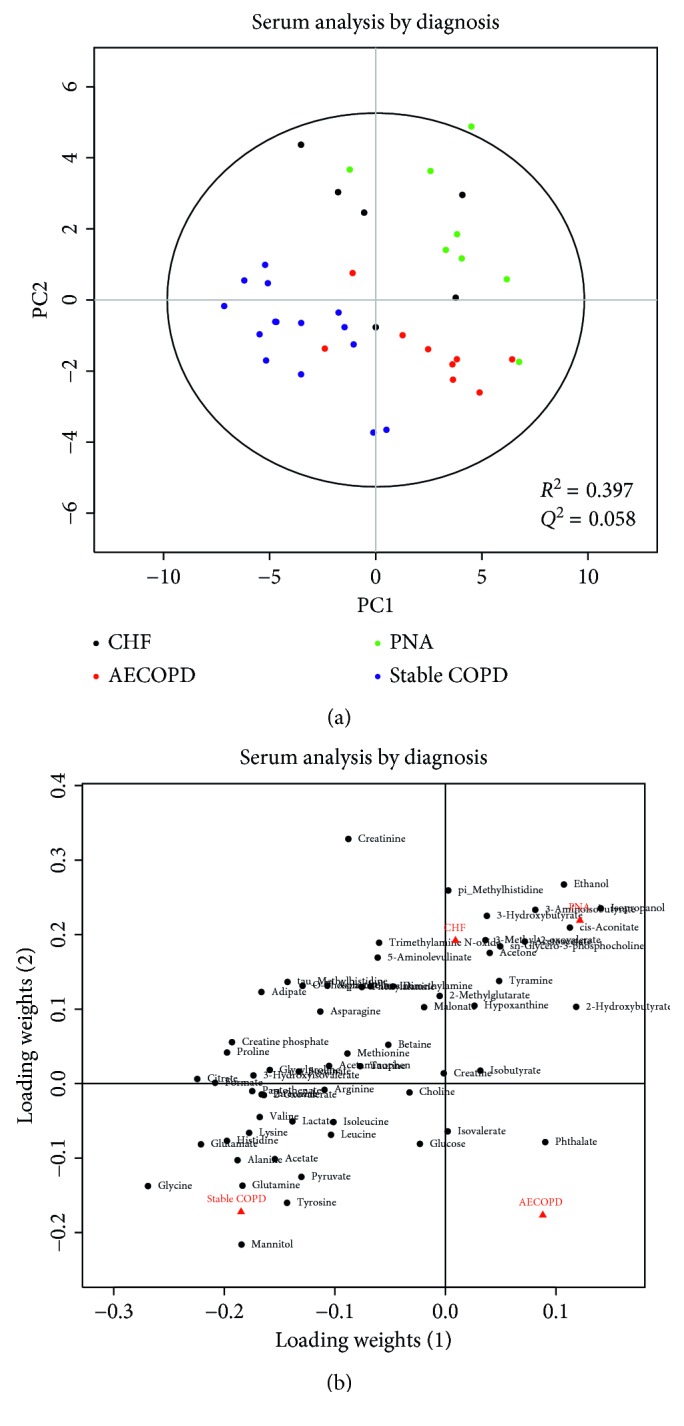
PLS-DA score plots (a) and loading plots (b) of serum samples drawn from patients with stable COPD (blue), AECOPD (red), CHF (black), or PNA (green). Each circle represents a serum sample. Some separation between the groups can be seen. The loading plot shows how the profiled metabolites contribute to the separation seen in the scores. The model is statistically significant (*p*=0.001), but the fit is moderate at best (*R*^2^ = 0.397) with poor predictive value (*Q*^2^ = 0.058). Taken with the rest of the results, our interpretation is that the separation is likely due to the presence of respiratory failure.

**Figure 2:**
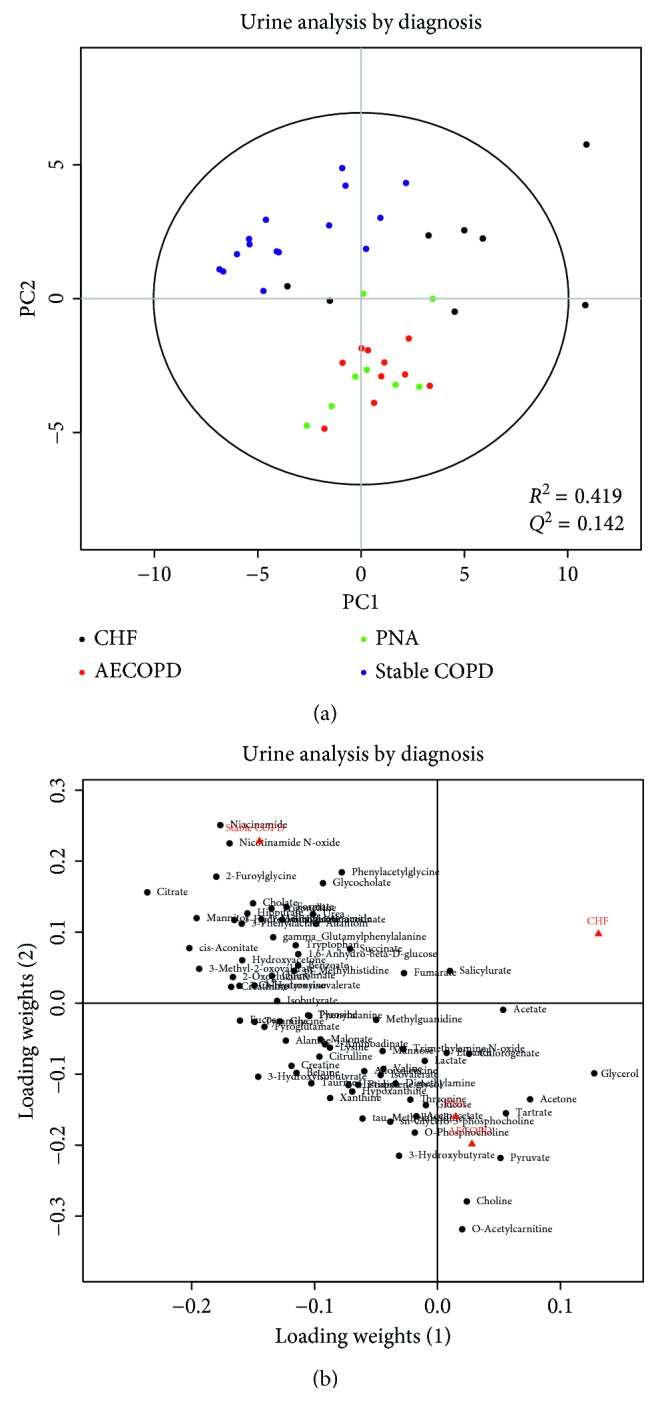
PLS-DA score plots (a) and loading plots (b) of urine samples drawn from patients with stable COPD (blue), AECOPD (red), CHF (black), or PNA (green). Each circle represents a urine sample. Some separation between the groups can be seen. The loading plot shows how the profiled metabolites contribute to the separation seen in the scores. The model is statistically significant (*p* < 0.001), but the fit is moderate at best (*R*^2^ = 0.419) with moderate predictive value (*Q*^2^ = 0.142). Taken with the rest of the results, our interpretation is that the separation is likely due to the presence of respiratory failure.

PLS-DA showed that serum metabolic profiles were significantly different among the various diagnosis groups (*p*=0.001, *R*^2^ = 0.397, *Q*^2^ = 0.058) (Figure 1). Similarly, we observed significant difference in the urine metabolic profiles of the various diagnostic groups (*p* < 0.001*R*^2^ = 0. 419, *Q*^2^ = 0.142) (Figure 2). Concentrations of the top 10 VIP (variable of importance in projection) metabolites are compared in both serum (Table 2) and urine (Table 3).

**Figure 3:**
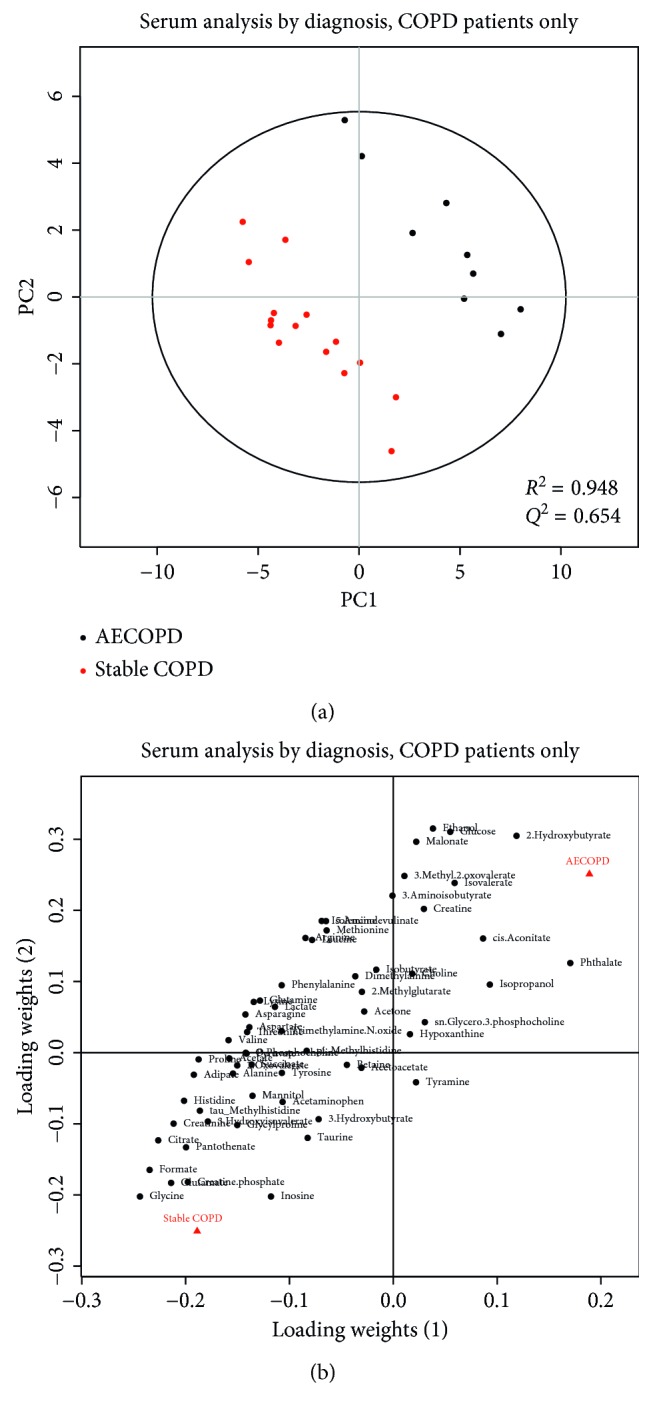
PLS-DA score plots (a) and loading plots (b) of serum samples drawn from patients with stable COPD (red) or AECOPD (black). Each circle represents a serum sample. The two groups are well separated in the score plots. The loading plot shows how the profiled metabolites contribute to the separation seen in the scores. The model is not statistically significant (*p*=0.997), but the fit is quite good (*R*^2^ = 0.948), as is the predictive value (*Q*^2^ = 0.654). This indicates that the model is overfit and suffers from a low sample size.

**Figure 4:**
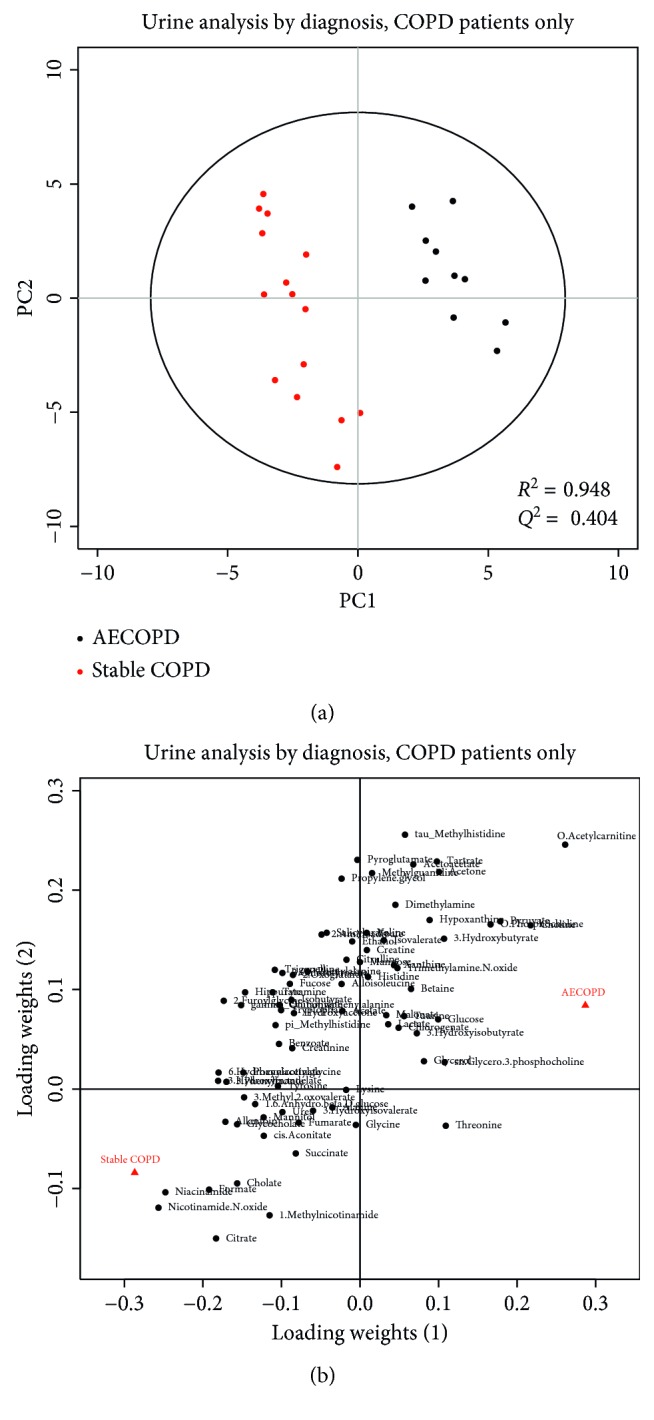
PLS-DA score plots (a) and loading plots (b) of urine samples drawn from patients with stable COPD (red) or AECOPD (black). Each circle represents a urine sample. The two groups are well separated in the score plots. The loading plot shows how the profiled metabolites contribute to the separation seen in the scores. The model is not statistically significant (*p*=0.929), but the fit is quite good (*R*^2^ = 0.948), as is the predictive value (*Q*^2^ = 0.404). This indicates that the model is overfit and suffers from a low sample size.

We also performed a metabolomic analysis limited to subjects with stable COPD and patients with AECOPD. Stable COPD subjects' profiles were not significantly different from patients with AECOPD in serum (*p*=0.997, *R*^2^ = 0.948, *Q*^2^ = 0.654) or urine (*p*=0.929, *R*^2^ = 0.948, *Q*^2^ = 0.404; Figures 3 and 4, resp.). At first glance, the score plots appear to distinguish AECOPD patients from stable COPD patients quite well and with good predictability (*R*^2^ > 0.9, *Q*^2^ > 0.4). However, the permutation *p* values for the models are highly insignificant (*p* > 0.9). This indicates that the model is overfit, which is likely due to small sample size. Further investigation with larger patient groups is warranted.

We then investigated whether the differences shown in the PLS-DA models in Figures 1 and 2 were related to the presence of respiratory failure only and not any specific diagnosis (e.g., AECOPD, CHF, or PNA). Thus we analyzed the metabolic profiles from all patients in respiratory failure, excluding the stable COPD group. These PLS-DA models showed similar metabolic profiles among the groups in both serum (*p*=0.2, *R*^2^ = 0.631, *Q*^2^ = 0.246) and urine (*p*=0.065, *R*^2^ = 0.602, *Q*^2^ = −0.134) (Supplemental Figures 1 and 2 in Supplementary Material available online at http://doi.org/10.1155/2017/9480346). Model parameters show at best a moderate fit with moderate (serum) to poor (urine) predictability. Neither model is statistically significant, though the urine model is close. These models may improve with larger patient groups.

We also analyzed serum and urine metabolic profiles for differences in smoking status, ICU length of stay, hospital length of stay, and duration of NIPPV (>4 days) and for differences in mortality and discharge destination. None of these models were statistically significant and thus could not differentiate serum or urine samples based upon these clinical indicators.

## 4. Discussion

In serum and urine metabolic profiling, COPD subjects with no exacerbation clustered separately from patients with respiratory failure requiring NIPPV due to AECOPD, CHF, or PNA (Figures 1 and 2). After exclusion of patients with stable COPD, the respiratory failure groups did not reliably cluster separately (Supplemental Figures 1 and 2). Our interpretation is that the observed differences in metabolic profiles in Figures 1 and 2 are due to whether the patient was in respiratory failure (CHF, AECOPD, and PNA) or not (stable COPD).

While our PLS-DA models comparing serum and urine samples from AECOPD patients with stable COPD patients (Figures 3 and 4) only showed good fit and predictability, the permutation *p* values of these models were not significant. Our findings are not consistent with those of a previous study that showed a distinct metabolic profile between patients with AECOPD and patients with stable COPD [13], likely due to our small sample size.

Despite the lack of a definitive global metabolic signature of AECOPD, we did find some potential markers that are worth further investigation. Serum glycine was decreased in AECOPD patients compared to the levels observed in subjects with stable COPD (Table 2). Glycine is a precursor to proteins and compromises 35% of collagen [17]. Serum glycine levels are inversely related with the degree of radiographic emphysema and cachexia in COPD patients [12]. Patients with AECOPD likely suffered from more advanced emphysema compared to the stable COPD subjects, but glycine levels were low in all type of respiratory failures (did not reach statistical significance for CHF) indicating another mechanism for the low glycine levels. Although the BMI was similar for all study groups, it is possible that the subjects with respiratory failure were more cachectic (reduced muscle mass) [12]. Glycine is also negatively correlated with inflammatory markers like C-reactive protein in chronic kidney disease [18] but in COPD patients, it is positively correlated [12]. Glycine is a molecule with anti-inflammatory properties, and exogenous administration reduces cytokine production [19].

Formate was also reduced in plasma of patients with respiratory failure. This is likely due to the fact that anabolism is impaired during acute illness. Formate is an intermediate product in normal metabolism [20].

Histidine, like glycine, is negatively correlated with radiographic emphysema [12]. Histidine is another amino acid with anti-inflammatory properties [21]. It is negatively associated with inflammation and oxidative stress [22], which is consistent with reduced levels in patients with AECOPD and PNA in our study. We observed the same pattern (reduced levels in AECOPD and PNA but not in the CHF group) in other metabolites: citrate, glutamate, proline, and creatine phosphate. Citrate and glutamate were reduced in AECOPD and PNA likely to generate energy [23, 24]. Serum proline was also reduced as a result of low glutamate levels [25]. Similarly, creatine phosphate, which serves as a rapid reserve energy storage, was decreased in AECOPD and PNA.

Respiratory failure secondary to AECOPD and PNA is a condition with higher inflammatory and metabolic state than respiratory failure due to CHF [26, 27], which is reflected on the specific metabolite analysis. The low levels of certain metabolites in AECOPD and PNA groups indicate that. However, we could not identify a single serum metabolite that could differentiate AECOPD from other causes of respiratory failure.

With the exception of citrate, the changes of the aforementioned metabolites did not occur in the urine. Urine citrate decreased in respiratory failure which reflects the high-demand metabolic state.

Furoylglycine levels were lower in the urine of patients with respiratory failure but they did not reach statistical significancy in the CHF and PNA groups. This finding is likely of no clinical importance as furoylglycine is an intermediated product of fatty acids and increases in certain inborn mitochondrial diseases [28] and after coffee consumption [29]. We observed the same pattern in the 3-hydroxymandelate levels. 3-Hydroxymandelate is a naturally occurring catecholamine metabolite which should be higher in the urine of patients with respiratory failure [30]. It is unclear why the 3-hydroxymandelate levels decreased in the urine of patients with respiratory failure.

Niacinamide and nicotinamide N-oxide were concentrated in very small amounts in urine and decreased in AECOPD and PNA. This likely reflects the reduced food intake that precedes AECOPD and PNA due to reduced appetite, and it is not present in CHF. As with serum, metabolomic analysis in urine did not reveal any specific AECOPD biomarker.

Even after stratification by duration of NIPPV, ICU and hospital LOS, and ICU mortality, metabolic profiling in both serum and urine showed no difference between patients requiring longer NIPPV versus patients requiring shorter NIPPV, between patients that stayed in the ICU or hospital for longer periods versus patients with shorter LOS, and in patients transferred out of the ICU alive versus patients that did not survive. These findings confirm that the clustering of the various respiratory failure groups did not result from the different degree of severity between the groups.

Although the metabolic state in respiratory failure is most likely the same regardless of the cause of the respiratory failure, the small sample size has likely contributed to the fact that we did not detect an AECOPD biomarker. Our sample size estimates were performed from a previous metabolomics-based COPD study available at the time, which compared COPD patients to healthy controls instead of patients with respiratory failure. Another limitation of our study is that we had spirometric data only for the stable COPD subjects and for a minority of AECOPD subjects. We did not include stable CHF patients and CHF or PNA patients that did not have respiratory failure. We also enrolled subjects in a convenient fashion, and there was low enrollment due to strict exclusion criteria. The subjects in our sample did suffer only from one of those 3 diseases (COPD, CHF, or PNA) while they usually coexist in respiratory failure patients. However, we wanted to ensure that the metabolic changes in AECOPD were purely due to COPD. Moreover, we collected the data prospectively, and a different investigator performed the spectrometry blindly. Our study strength is also that we focused only on patients with respiratory failure requiring NIPPV decreasing the chance to have included overdiagnosed AECOPD patients.

In conclusion, serum and urine metabolites clustered separately in subjects with stable COPD and patients with respiratory failure requiring NIPPV due to AECOPD, CHF, or PNA. However, we could not find a biomarker unique to AECOPD diagnosis. Despite this, levels of certain metabolites changed in conditions with high metabolic state like AECOPD and PNA. Further studies with larger sample sizes should investigate metabolic biomarkers which can be used in early diagnosis of AECOPD.

## Supplementary Material

Serum and urine metabolic profiles in patients with respiratory failure.

## Figures and Tables

**Table 1:** Subjects characteristics.

	Stable COPD (*n* = 15)	AECOPD (*n* = 12)	CHF (*n* = 8)	PNA (*n* = 9)
Age, *y* ± SD	68 ± 10.1	73.1 ± 10.6	78.5 ± 9.1	65.7 ± 17.3
Female, % (*n*)	60% (9)	66.6% (8)	62.5% (5)	33.3% (3)
BMI, kg/m^2^ ± SD	29.25 ± 5.7	28.8 ± 5.3	29.1 ± 8.6	29.8 ± 9.5
FEV1% predicted	44.7 ± 13.3	47.4 ± 10.6^∗^	NA	NA
Duration of NIPPV, *d* ± SD	NA	4.9 ± 3.4	1.8 ± 0.8	7.2 ± 4.7
Intubation, % (*n*)	NA	8.3% (1)	25% (2)	11.1% (1)
ICU LOS, *d* ± SD	NA	5 ± 3.9	6 ± 8.6	6.2 ± 5
ICU mortality, % (*n*)	NA	8.3% (1)	25% (2)	33.3% (3)
Hospital LOS, *d* ± SD	NA	6.1 ± 4.1	8 ± 8.8	9.8 ± 4.3
Hospital mortality, % (*n*)	NA	8.3% (1)	25% (2)	33.3% (3)
Discharge home, % (*n*)	NA	66.6% (8)	50% (4)	33.3% (3)

^∗^Available FEV1% predicted only for 6 subjects. AECOPD = acute exacerbation of COPD, BMI = body mass index, CHF = heart failure, LOS = length of stay, NIPPV = noninvasive positive pressure ventilation, PNA = pneumonia.

**Table 2:** Concentration (millimolar) of the top 10 VIP metabolites in serum of subjects with stable COPD, AECOPD, CHF, and PNA.

	Stable COPD (*n* = 15)	AECOPD (*n* = 12)	CHF (*n* = 8)	PNA (*n* = 9)
Glycine	3.9 × 10^−1^ ± 2.8 × 10^−1^^∗^^‡^	1.6 × 10^−2^ ± 2.3 × 10^−2^	3.3 × 10^−2^ ± 2.8 × 10^−2^	5 × 10^−3^ ± 1.8 × 10^−3^
Glutamine	7.2 × 10^−2^ ± 1.9 × 10^−2‡^	5.4 × 10^−2^ ± 1.7 × 10^−2^	6.1 × 10^−2^ ± 2.9 × 10^−2^	3.2 × 10^−2^ ± 1.6 × 10^−2^
Alanine	4.4 × 10^−2^ ± 1.8 × 10^−2‡^	2.8 × 10^−2^ ± 1.3 × 10^−2^	3.6 × 10^−2^ ± 2.3 × 10^−2^	1.6 × 10^−2^ ± 7.3 × 10^−3^
Proline	3.3 × 10^−2^ ± 1.3 × 10^−2^^∗^^‡^	1.7 × 10^−2^ ± 6.2 × 10^−3^	3.2 × 10^−2^ ± 1.4 × 10^−2‡^	1.3 × 10^−2^ ± 6.3 × 10^−3^
Glutamate	2.1 × 10^−2^ ± 7.6 × 10^−3^^∗^^‡^	8.6 × 10^−3^ ± 5.4 × 10^−3^	1.3 × 10^−2^ ± 8.7 × 10^−3^	6.8 × 10^−3^ ± 4.1 × 10^−3^
Mannitol	1.5 × 10^−2^ ± 10^−2†‡^	4.9 × 10^−3^ ± 2.9 × 10^−3^	3.1 × 10^−3^ ± 1.7 × 10^−3^	2.8 × 10^−3^ ± 1.4 × 10^−3^
Citrate	1.2 × 10^−2^ ± 7 × 10^−3^^∗^^‡^	4.2 × 10^−3^ ± 1.7 × 10^−3^	7.4 × 10^−3^ ± 2.9 × 10^−3^	4.4 × 10^−3^ ± 2.1 × 10^−3^
Histidine	9.2 × 10^−3^ ± 2.8 × 10^−3^^∗^^‡^	4.7 × 10^−3^ ± 2.5 × 10^−3^	5.9 × 10^−3^ ± 4.3 × 10^−3^	4.1 × 10^−3^ ± 3.4 × 10^−3^
Formate	6.2 × 10^−3^ ± 3.5 × 10^−3^^∗^^†‡^	1.6 × 10^−3^ ± 6.5 × 10^−4^	2 × 10^−3^ ± 6.8 × 10^−4^	2.8 × 10^−3^ ± 1.6 × 10^−3^
Creatine phosphate	3.6 × 10^−3^ ± 1.9 × 10^−3^^∗^^‡^	1.5 × 10^−3^ ± 2.9 × 10^−4†^	3.2 × 10^−3^ ± 1.310^−3‡^	1.6 × 10^−3^ ± 5.2 × 10^−4^

^∗^ *p* < 0.05 versus AECOPD, ^†^*p* < 0.05 versus CHF, ^‡^*p* < 0.05 versus PNA. AECOPD = acute exacerbation of COPD, CHF = congestive heart failure, PNA = pneumonia.

**Table 3:** Concentration (millimoles of metabolite per millimoles of total solute) of the top 10 VIP metabolites in urine of subjects with stable COPD, AECOPD, CHF, and PNA.

	Stable COPD (*n* = 15)	AECOPD (*n* = 12)	CHF (*n* = 8)	PNA (*n* = 9)
Creatinine	1.4 × 10^−2^ ± 8.6 × 10^−3^	1 × 10^−2^ ± 4 × 10^−3^	7.3 × 10^−3^ ± 5.6 × 10^−3^	9.1 × 10^−3^ ± 3.5 × 10^−3^
Citrate	2.9 × 10^−3^ ± 1.9 × 10^−3^^∗^^†‡^	1.7 × 10^−3^ ± 3 × 10^−3^	4.8 × 10^−4^ ± 8.8 × 10^−4^	6.1 × 10^−4^ ± 5.2 × 10^−4^
Mannitol	2.6 × 10^−3^ ± 2.3 × 10^−3†^	1.2 × 10^−3^ ± 1.1 × 10^−3^	4.2 × 10^−4^ ± 3.1 × 10^−4^	8.5 × 10^−4^ ± 7.6 × 10^−4^
*Cis*-aconitate	5.6 × 10^−4^ ± 2.5 × 10^−4†^	4 × 10^−4^ ± 2.6 × 10^−4^	2.6 × 10^−4^ ± 3.3 × 10^−4^	2.8 × 10^−4^ ± 1.1 × 10^−4^
Furoylglycine	3.7 × 10^−4^ ± 5.3 × 10^−4^^∗^	7 × 10^−5^ ± 8.7 × 10^−5^	6.6 × 10^−5^ ± 8.4 × 10^−5^	4.7 × 10^−5^ ± 3.5 × 10^−5^
3-Hydroxymandelate	3 × 10^−4^ ± 2.710^−4^^∗^	8.5 × 10^−5^ ± 7.2 × 10^−5^	1.3 × 10^−4^ ± 1.7 × 10^−4^	1.1 × 10^−4^ ± 1.1 × 10^−4^
Oxoglutarate	3 × 10^−4^ ± 1.910^−4†^	2 × 10^−4^ ± 1.2 × 10^−4^	1.4 × 10^−4^ ± 1.5 × 10^−4^	1.3 × 10^−4^ ± 5.6 × 10^−5^
Methyl-2-oxovalerate	1.1 × 10^−4^ ± 8.2 × 10^−5†^	5.9 × 10^−5^ ± 3.2 × 10^−5^	4.1 × 10^−5^ ± 4.210^−5^	6.5 × 10^−5^ ± 5.5 × 10^−5^
Nicotinamide N-oxide	2.5 × 10^−5^ ± 4 × 10^−5^^∗^^‡^	2.9 × 10^−6^ ± 6.2 × 10^−6^	7 × 10^−6^ ± 1.1 × 10^−5^	6.2 × 10^−6^ ± 1.3 × 10^−5^
Niacinamide	1.7 × 10^−5^ ± 1.1 × 10^−5‡†^	2.2 × 10^−6^ ± 3.5 × 10^−6^	5.2 × 10^−6^ ± 6.9 × 10^−6^	1.8 × 10^−6^ ± 3.1 × 10^−6^

We selected the top-10 metabolites with the highest VIP score. ^∗^*p* < 0.05 versus AECOPD, ^†^*p* < 0.05 versus CHF, ^‡^*p* < 0.05 versus PNA. AECOPD = acute exacerbation of COPD, CHF = congestive heart failure, PNA = pneumonia.

## References

[1] CDC (2012). Chronic obstructive pulmonary disease among adults—United States, 2011. *Morbidity and Mortality Weekly Report*.

[2] Darnell K., Dwivedi A. K., Weng Z., Panos R. J. (2013). Disproportionate utilization of healthcare resources among veterans with COPD: a retrospective analysis of factors associated with COPD healthcare cost. *Cost Effectiveness and Resource Allocation*.

[3] Sullivan S. D., Ramsey S. D., Lee T. A. (2000). The economic burden of COPD. *Chest*.

[4] Strassels S. A., Smith D. H., Sullivan S. D., Mahajan P. S. (2001). The costs of treating COPD in the United States. *Chest*.

[5] Nannini L. J. (2012). Hospitalization due to COPD exacerbation. *Chest*.

[6] Connors A. F., Dawson N. V., Thomas C. (1996). Outcomes following acute exacerbation of severe chronic obstructive lung disease. The SUPPORT investigators (Study to Understand Prognoses and Preferences for Outcomes and Risks of Treatments). *American Journal of Respiratory and Critical Care Medicine*.

[7] Bourbeau J., Ford G., Zackon H., Pinsky N., Lee J., Ruberto G. (2007). Impact on patients’ health status following early identification of a COPD exacerbation. *European Respiratory Journal*.

[8] Wilkinson T. M. A., Donaldson G. C., Hurst J. R., Seemungal T. A. R., Wedzicha J. A. (2004). Early therapy improves outcomes of exacerbations of chronic obstructive pulmonary disease. *American Journal of Respiratory and Critical Care Medicine*.

[9] Bourbeau J., Sedeno M. F., Metz K., Li P. Z., Pinto L. (2016). Early COPD exacerbation treatment with combination of ICS and LABA for patients presenting with mild-to-moderate worsening of dyspnea. *COPD: Journal of Chronic Obstructive Pulmonary Disease*.

[10] Laue J., Reierth E., Melbye H. (2015). When should acute exacerbations of COPD be treated with systemic corticosteroids and antibiotics in primary care: a systematic review of current COPD guidelines. *NPJ Primary Care Respiratory Medicine*.

[11] Wang L., Tang Y., Liu S. (2013). Metabonomic profiling of serum and urine by ^1^H NMR-based spectroscopy discriminates patients with chronic obstructive pulmonary disease and healthy individuals. *PLoS One*.

[12] Ubhi B. K., Riley J. H., Shaw P. A. (2012). Metabolic profiling detects biomarkers of protein degradation in COPD patients. *European Respiratory Journal*.

[13] Gulcev M., Reilly C., Griffin T. J. (2016). Tryptophan catabolism in acute exacerbations of chronic obstructive pulmonary disease. *International Journal of Chronic Obstructive Pulmonary Disease*.

[14] Fortis S., Weinert C., Bushinski R., Koehler A. G., Beilman G. (2014). A health system-based critical care program with a novel tele-ICU: implementation, cost, and structure details. *Journal of the American College of Surgeons*.

[15] Beckonert O., Keun H. C., Ebbels T. M. (2007). Metabolic profiling, metabolomic and metabonomic procedures for NMR spectroscopy of urine, plasma, serum and tissue extracts. *Nature Protocols*.

[16] Lusczek E. R., Paulo J. A., Saltzman J. R. (2013). Urinary ^1^H-NMR metabolomics can distinguish pancreatitis patients from healthy controls. *JOP: Journal of the Pancreas*.

[17] TMIC (2005). http://www.hmdb.ca/metabolites/HMDB00123.

[18] Suliman M. E., Qureshi A. R., Stenvinkel P. (2005). Inflammation contributes to low plasma amino acid concentrations in patients with chronic kidney disease. *American Journal of Clinical Nutrition*.

[19] Wheeler M. D., Ikejema K., Enomoto N. (1999). Glycine: a new anti-inflammatory immunonutrient. *Cellular and Molecular Life Sciences*.

[20] TMIC (2005). http://www.hmdb.ca/metabolites/hmdb00142.

[21] Feng R. N., Niu Y. C., Sun X. W. (2013). Histidine supplementation improves insulin resistance through suppressed inflammation in obese women with the metabolic syndrome: a randomised controlled trial. *Diabetologia*.

[22] Watanabe M., Suliman M. E., Qureshi A. R. (2008). Consequences of low plasma histidine in chronic kidney disease patients: associations with inflammation, oxidative stress, and mortality. *American Journal of Clinical Nutrition*.

[23] Costello L. C., Franklin R. B. (2005). ‘Why do tumour cells glycolyse?’: from glycolysis through citrate to lipogenesis. *Molecular and Cellular Biochemistry*.

[24] Mates J. M., Segura J. A., Campos-Sandoval J. A. (2009). Glutamine homeostasis and mitochondrial dynamics. *The International Journal of Biochemistry & Cell Biology*.

[25] TMIC (2005). http://www.hmdb.ca/metabolites/hmdb00162.

[26] Pinto-Plata V. M., Livnat G., Girish M. (2007). Systemic cytokines, clinical and physiological changes in patients hospitalized for exacerbation of COPD. *Chest*.

[27] Schutte H., Lohmeyer J., Rosseau S. (1996). Bronchoalveolar and systemic cytokine profiles in patients with ARDS, severe pneumonia and cardiogenic pulmonary oedema. *European Respiratory Journal*.

[28] TMIC (2005). http://www.hmdb.ca/metabolites/HMDB00439.

[29] Heinzmann S. S., Holmes E., Kochhar S., Nicholson J. K., Schmitt-Kopplin P. (2015). 2-furoylglycine as a candidate biomarker of coffee consumption. *Journal of Agricultural and Food Chemistry*.

[30] TMIC (2005). http://www.hmdb.ca/metabolites/HMDB00750.

