# Supplementary material for: Metabolomics in COPD Acute Respiratory Failure Requiring Noninvasive Positive Pressure Ventilation
Source: Can Respir J. 2017 Dec 17;2017:9480346. doi: 10.1155/2017/9480346 (PMC5748128; doi:10.1155/2017/9480346)

Supplemental Figure 1: PLS-DA scores plots (left) and loadings plots (right) of serum samples drawn from patients with AECOPD (red), CHF (black), or PNA (green). Each circle represents a serum sample. Some separation between the groups can be seen. The loadings plot shows how the profiled metabolites contribute to the separation seen in the scores. The model is not statistically significant (p=0.2) and the fit is moderate (R^2^=0.631) with moderate predictive value (Q^2^=0.246). Taken with the rest of the results, our interpretation is that the metabolic effects of respiratory failure are not unique to diagnosis.


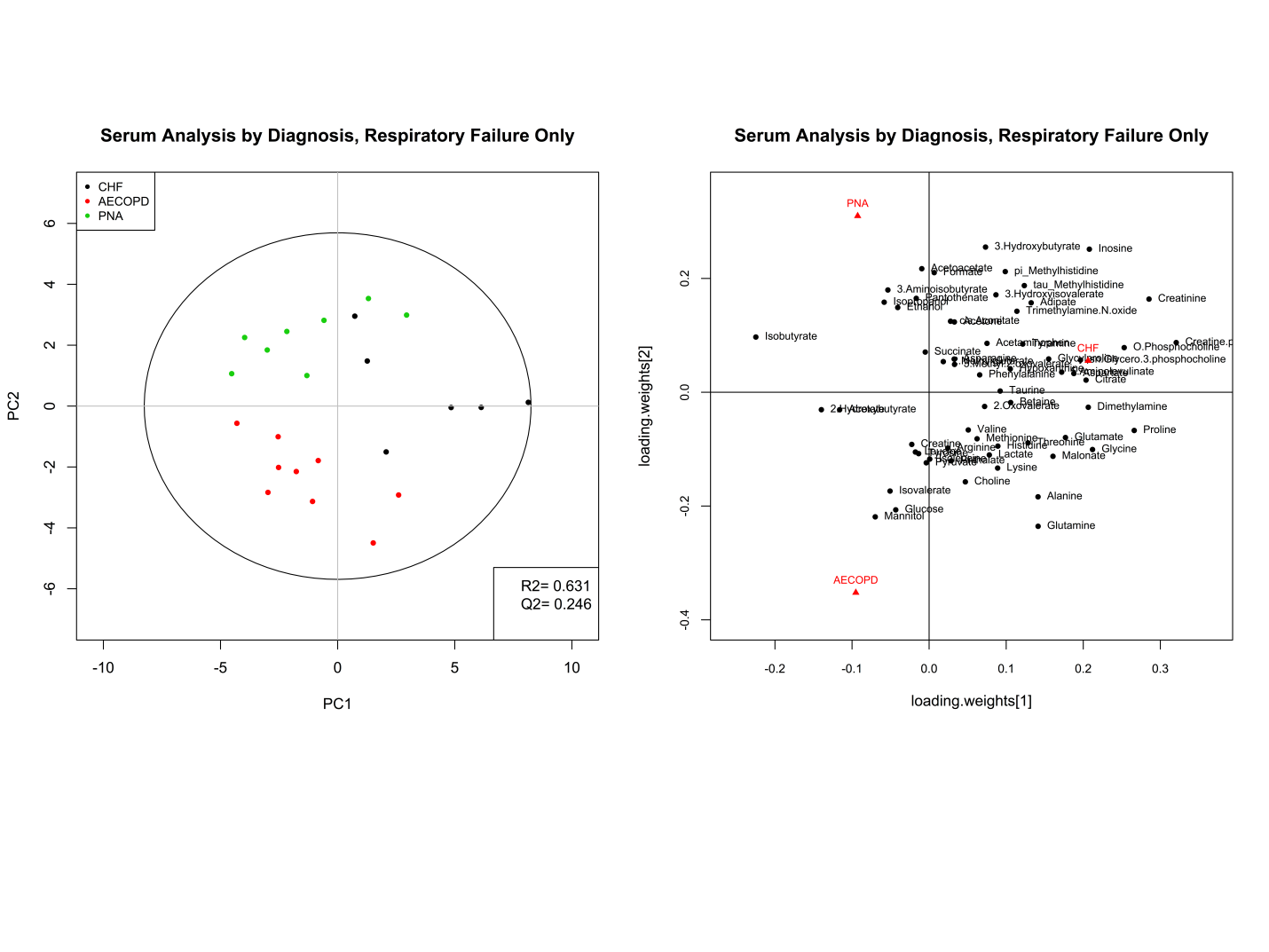


Supplemental Figure 2: PLS-DA scores plots (left) and loadings plots (right) of urine samples drawn from patients with AECOPD (red), CHF (black), or PNA (green). Each circle represents a urine sample. Some separation between the groups can be seen. The loadings plot shows how the profiled metabolites contribute to the separation seen in the scores. The model is nearly statistically significant (p=0.065) but the fit is moderate at best (R^2^=0.602) with no predictive value (Q^2^=-0.134). Taken with the rest of the results, our interpretation is that the metabolic effects of respiratory failure are not unique to diagnosis.


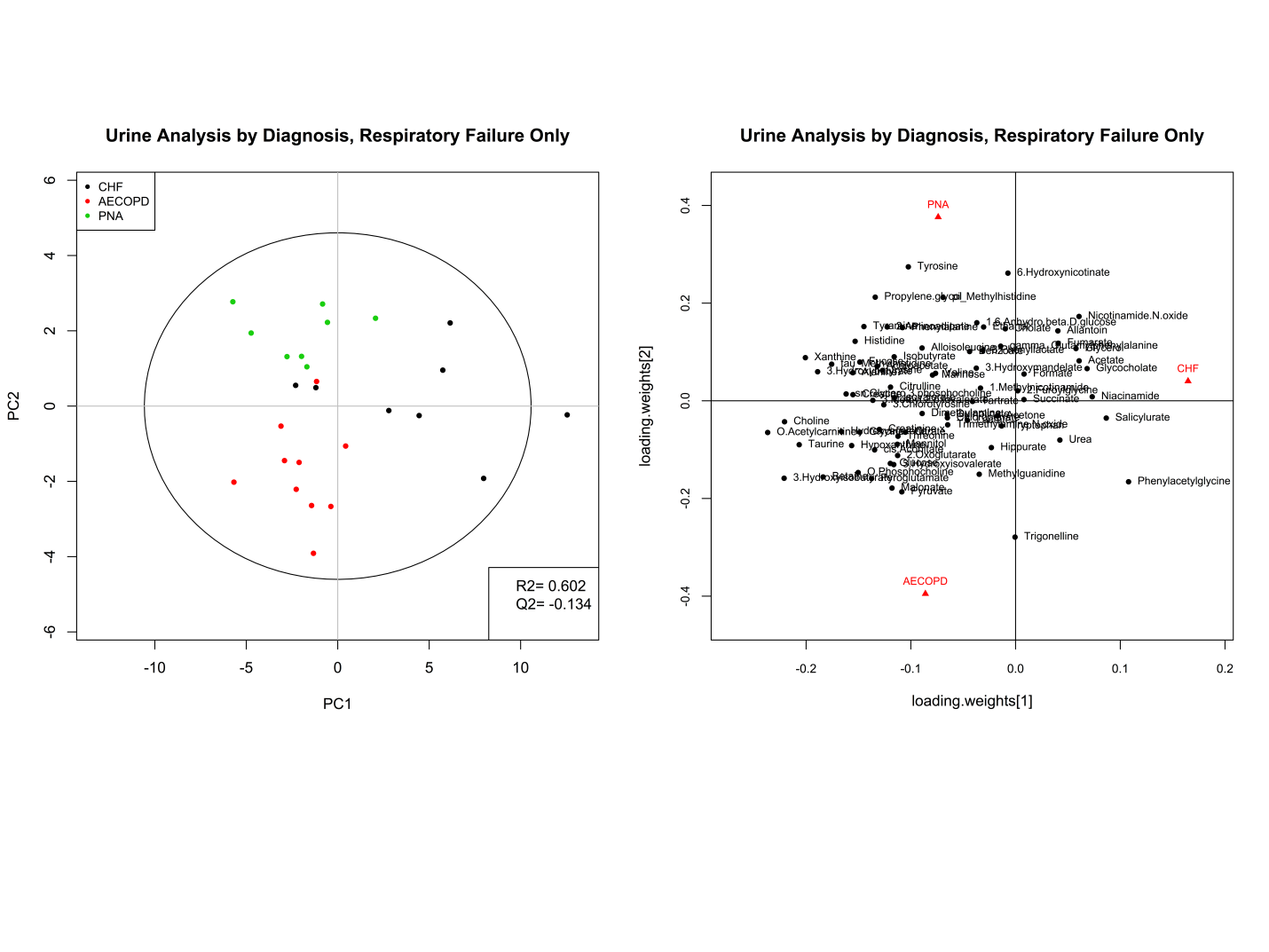

Supplement: Supplementary file 1 — Serum and urine metabolic profiles in patients with respiratory failure. [file 9480346.f1.docx]
